# Burden of shoulder and/neck pain among school teachers in Ethiopia

**DOI:** 10.1186/s12891-019-2397-3

**Published:** 2019-01-10

**Authors:** Melaku Hailu Temesgen, Gashaw Jember Belay, Asmare Yitayeh Gelaw, Balamurugan Janakiraman, Yaregal Animut

**Affiliations:** 1 Department of Physiotherapy, School of Medicine, College of Medicine and Health Sciences University of Gondar, P. O. Box: 196, Gondar, Ethiopia; 2 Department of Epidemiology and Preventive Medicine, Monash University, Melbourne, Victoria Australia; 3 Department of Epidemiology and Biostatistics, Institute of Public Health, College of Medicine and Health Sciences, University of Gondar, Gondar, Ethiopia

**Keywords:** Shoulder and/neck pain, School teachers, Posture, Hypertension, Ethiopia

## Abstract

**Background:**

Shoulder and neck pain are reported as the most common occupational-related health problem and cause of morbidity, absenteeism from work among school teachers worldwide. School teachers represent an occupational group, who are exposed and appears to have prevalent shoulder and/or neck pain due to their daily work tasks and the nature of work. There is a scant epidemiological study regarding shoulder and neck pain among school teachers in Ethiopia. Therefore, this study was set out to assess the prevalence and associated factors of shoulder and/or neck pain among school teachers of Gondar town in North West Ethiopia.

**Method:**

An institutional based cross-sectional study was conducted from December 2016 to January 2017, a structured questionnaire adapted from the Nordic musculoskeletal questionnaire was distributed to 848 primary and secondary school teachers in Gondar town, Northwest Ethiopia. To assess the burden of shoulder and/neck pain, data were collected using a self-administered questionnaire and physical measures like height and weight were also measured during data collection. Independent variables which had significant association were identified using logistic regression model.

**Result:**

A total of 754 teachers participated, with a mean age of 42 ± 9.73 years (88.9% response rate). Previous 12 months self-reported prevalence of shoulder and/ neck pain among school teachers was 57.3% with 95%CI (53.4–61.0%). Regular physical exercise (OR = 0.18, 95% CI: 0.08–0.42), teaching experience (OR = 2.85, 95% CI: 1.09–7.42), static head down posture (OR = 2.26, 95% CI: 1.55–3.33), elevated arm over shoulder (OR = 2.71, 95% CI: 1.86–3.95), prolonged sitting (OR = 1.50,95% CI: 1.02–2.23) and hypertension (OR = 2.18, 95% CI: 1.24–3.82) were factors found to be significantly associated with shoulder and/neck pain.

**Conclusion and recommendation:**

More than half of the study participants self-reported to have suffered shoulder and neck pain in the previous 12 months. Teaching experience, static head down posture, elevated arm over shoulder, and hypertension are likely to be significantly associated with shoulder and/ neck pain among school teachers in Ethiopia. Engaging in regular physical exercise has a protective effect against the shoulder and/or neck pain. Therefore, school authorities are recommended to provide facilities to enhance physical activity among school teachers and also provide adjustable board and classroom materials.

**Electronic supplementary material:**

The online version of this article (10.1186/s12891-019-2397-3) contains supplementary material, which is available to authorized users.

## Background

Among all the occupational-related health problems, pain in the shoulder and neck is the most common cause of morbidity and absenteeism from work in many countries [1–3] . From the general musculoskeletal disorders (MSD), shoulder-neck pain is the specific and particular pain among the different working population with its multifactorial bio-psychological origin and socio-economic costs [4–8].

School teachers represent an occupational group, which are exposed and appears to be at the risk of suffering shoulder and/or neck pain (SNP) due to their daily work tasks [4] . In a single day, a wide variety of duties and responsibilities may be carried out by school teachers which involve significant use of head down postures, such as prepare lessons, frequent reading, assessing/marking students and writing on blackboard under unfavorable working conditions, especially in low-middle income countries (LMIC’s) [3, 4, 9, 10] . While performing such daily tasks repeatedly for a long period of time using abnormal posture; they might develop pain or discomfort around shoulder/neck body segments [11] .

A systematic review conducted on “musculoskeletal disorders among teachers” from 13 countries(3- from America, 4- from Europe, 5- from Asia and Australia) reported that the prevalence of MSD among school teachers was between 40 and 90% [11] . A study in China reported that 12-month neck and/or shoulder pain prevalence among school teachers was 66.7% [12]. Upper back, shoulder, and neck MSD were common and reported at similar rates 52.6, 52.5,and 50.8% respectively in a cross-sectional study of Botswana school teachers [9].

Socio-demographic factors such as gender, age, body mass index (BMI), individual factors like smoking and drinking habit, physical exercise, work-related factors for instance working hours, head down posture, overhead activities and psycho-social factors like poor colleagues and supervisor support, low job satisfaction are considered as associated factors of shoulder and/neck musculoskeletal pain (MSP) among school teachers in different epidemiological studies [13–17].

Due to this vague pain, school teachers suffer a low quality of life, frequent sick leave, functional impairments, absenteeism, early retirement, disability and health cost. Our extensive search revealed scarce published regional works reporting the prevalence of SNP among school teachers in the sub-Saharan region and none were found in Ethiopia. Hence, the aim of this study was to determine the prevalence and identify factors associated with SNP among primary and secondary school teachers in North West Ethiopia.

## Method

### Study design, period and study area

An institutional based cross-sectional study was conducted in Gondar town, Northwest Ethiopia from December 8, 2016, to January 30, 2017. Gondar town is a historical terrain in the country and located at 727 km Northwest of Addis Ababa, the capital city of Ethiopia. According to the 2007 Ethiopian census report, Gondar has 206,987 total population [18]. Administratively the town is divided into 12 administrative areas. It has one University, 2 Colleges, 1 Technical and Vocational school, 14 secondary schools and 65 primary schools. The total numbers of teachers working in primary and secondary schools of both private and governmental schools of Gondar town were about 1500 [19].

### Source and study population

All school teachers working in both primary (1–8 grade) and secondary (9–10 grade) schools of private and Governmental institutions in Gondar town were used as source population and Randomly selected teachers who were working in the proportionally nominated schools were study subjects.

### Sample size and sampling method

The sample size of the study was calculated using a single population proportion formula by considering 50% prevalence of SNP, 95% confidence interval and 5% margin of error. Finally, the sample size of 848 was obtained by addition of 10% non-response rate, and a design effect of 2.

Multi-stage sampling technique was employed to select the study participants. First, Schools in the town were stratified into primary and secondary schools based on the level of the institution. Then each stratum was further classified into private and governmental schools. Subsequently, 22 schools were proportionally selected using simple random sampling from each stratum. From the selected schools the samples were proportionally recruited based on the total number of teachers in that school using an alphabetical list obtained.

### Data collection procedures and quality control

A structured self-administered questionnaire (Additional file 1) was distributed to collect the data. The questionnaire was first prepared in English language and then translated to Amharic (local language) and again back to English by language experts and was checked for the consistency. Tools used to assess shoulder/neck pain was adapted from the standardized Nordic questionnaire and modified to local context [20]. Before the actual data collection a pre-test was carried out with 10% of power calculated sample out of the study area. Intensive training was provided to data collectors and supervisors for 2 days. Six physiotherapists were recruited to distribute the self-administered questionnaire (including the written consent form with a single page in front of the questionnaire), to assemble the filled questionnaire, and take physical measures. Weight was measured using floor weighing scale (Electrolux, Korea) with participants standing without shoes and wearing light clothing and recorded to the nearest 0.5Kg. Height was measured using stadiometer at standing upright with the head in the Frankfort plane and recorded with an approximation of 1 cm. The data collection process was closely monitored by the principal investigator (MH) and the supervisors throughout the data collection period. Filled questionnaires were checked daily for completeness of information and conflicts were reported to data collectors.

### Data analysis

Data was coded and entered into Epi info software version 7.0 and analyzed using IBM Statistical Package for Social Sciences (SPSS) version 23 for windows. The results were presented using text, frequency distribution tables, and graph for descriptive statistics. Logistics regression model was used to identify factors associated with shoulder and/or neck pain. Independent variables with a *p*-value less than < 0.2 in the bivariate logistic regression were fitted into the multivariable logistic regression analysis for controlling the possible main effect of confounders and interaction terms was used to examine the potential associations.. Results were considered statistically significant when 95% confidence intervals not containing unity (equal to *p-*value < 0.05) for both main effects and interaction terms.

### Operational definitions

**Burden**: it is defined as the prevalence of shoulder and neck pain.

**Shoulder and/ neck pain**: ache, pain, or discomfort felt at a time in the shoulder and neck (cervico-brachial region) in the last 12 months.

## Result

### Socio-demographic characteristics

A total of 848 questionnaires were distributed, of which 754 teachers responded hence the response rate was 88.9%. Majority of the study participants (57.8%) were females. The mean age of the respondents was 42 years (SD ± 9.732 years). Nearly, one-third of them (32.8%), were in the age group of 31–40 years. The majority of teachers were from primary schools and governmental schools 77.5 and 83.4% respectively (Table 1).

**Table 1:** Socio-demographic characteristics of school teachers in Gondar town January, 2017 (*N*=754)

Variables	Frequency = N	Percent = %
Sex		
Male	318	42.2%
Female	436	57.8%
Pregnant		
Yes	14	3.2%
No	422	96.8%
Age		
<31	119	15.8%
31-40	246	32.8%
41-50	214	28.5%
>50	175	22.9%
Marital status		
Single	96	12.7%
Married	624	82.8%
Others	34	4.5%
Educational level		
Certificate	9	1.2%
Diploma	387	51.3%
Degree	346	45.9%
Master	12	1.6%
Salary		
2000-4000	155	20.6%
4001-6000	517	68.6%
>6001	82	10.9%
Handedness		
Right	734	97.3%
Left	20	2.7%
School		
Primary	584	77.5%
Secondary	170	22.5%
Organization		
Private	125	16.6%
Public	629	83.4%

### Behavioral and physical characteristics

**Fig. 1:**
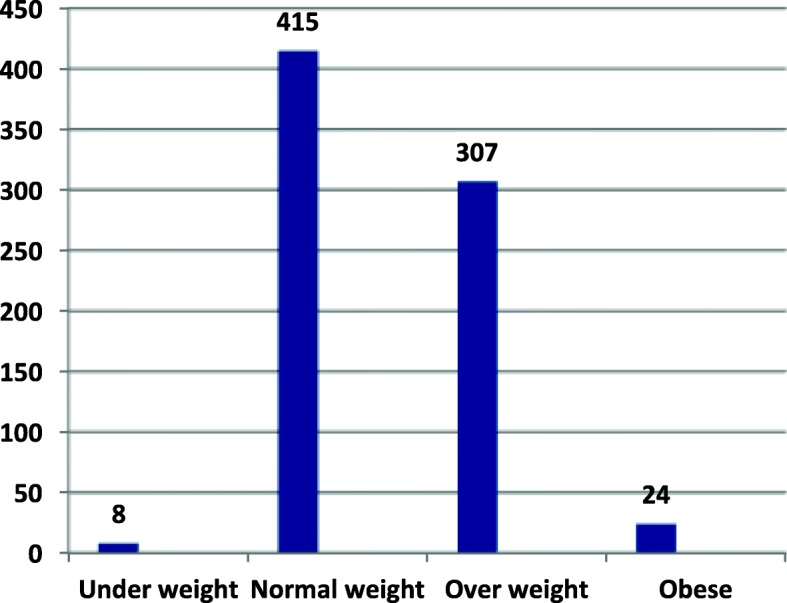
This figure is a bar graph for body mass index categories of school teachers in Gondar town, Ethiopia 2017 (*N*=754)

Only 3.2% of the teachers were current smokers and 7.7% were previous smokers. A minority of the respondents (7.4%) were having a habit of drinking alcohol. A small proportion of (7.2%) participants were involved in regular physical exercises for more than 150 min per week. More than half (55%) of the participant’s BMI was categorized as normal weight followed by overweight (Fig. 1).

### Work -related characteristics and comorbidities

More than one-thirds of the teachers (37.4%) have 10–19 years of teaching experience with a overall mean of 18.79 ± 10.24 years. One hundred thirty-nine (18.4%) of respondents reported being involved in more than 30 h of teaching in a week. The majority (70%), of teachers, indicated that they were using static head down posture and elevated arm over the shoulder (69.4%) for more than 2 h in a day while they were teaching students. Among the participants 15 and 17% of school teachers self-reported to have been diagnosed with diabetes and hypertension respectively (Table 2).

**Table 2:** Work related characteristics and comorbidities of school teachers in Gondar town, January, 2017 (*N*=754)

Variables	Frequency N (%)	Percent =%
Teaching experience (year)		
2-9	151	20.0%
10-19	282	37.4%
20-29	163	21.6%
>=30	158	21.0%
Teaching hours/ week		
<=30	615	81.6%
>30	139	18.4%
Number of students in class		
21-30	42	5.6%
31-40	129	17.1%
>40	564	77.3%
Static head down posture(>2hr/day)		
Yes	528	70.0%
No	226	30.0%
Elevated arm (>2hr/day)		
Yes	523	69.4%
No	231	30.6%
Prolonged sitting (>4hr./day)		
Yes	247	32.8%
No	507	67.2%
Comfortable upper back support		
Yes	134	17.8%
No	620	82.2%
Diabetes mellitus		
Yes	113	15.0%
No	641	85.0%
Hypertension		
Yes	128	17.0%
No	626	83.0%
Respiratory diseases		
Yes	65	8.6%
No	689	91.4%
Previous history of surgery		
Yes	12	1.6%
No	742	98.4%
Previous history of Trauma		
Yes	35	4.6%
No	719	95.4%

Regarding psychosocial characteristics, 281 (37.1%) of them were satisfied by their profession and 290 (38.5%) of school teachers were sometimes supported by their supervisors (Table 3).

**Table 3:** Psychosocial characteristics of school teachers in Gondar town, 2017 (*n*=754)

Variables	Frequency=N	Percent = %
Supervisor support		
Always	45	6.0%
Often	208	27.6%
Sometimes	290	38.5%
Seldom	150	19.9%
Never	61	8.1%
Social support		
Always	13	1.7%
Often	79	10.5%
Sometimes	217	28.8%
Seldom	232	30.8%
Never	213	28.2%
Co-worker support		
Always	39	5.2%
Often	196	26.0%
Sometimes	270	18.0%
Seldom	136	15.0%
Never	113	18.7%
Job satisfaction		
Highly satisfied	66	37.1%
Satisfied	281	25.9%
Neutral	263	11.3%
Unsatisfied	90	7.0%
Highly unsatisfied	54	6.0%

### Prevalence and distribution of shoulder and/neck pain among school teaches

Previous 12 months self-reported prevalence of shoulder and/ neck pain among school teachers was 57.3% (95% CI: 53.4–61.0%). The prevalence of SNP was higher among female teachers, primary public school teachers, teachers who have more than 30 years of teaching experiences 59.7, 60.42 and 77.2%) respectively. Teachers with body mass index ≥30 reported higher (91.6%) prevalence of shoulder and/ or neck pain.

### Factors associated with shoulder and/neck pain

In the bivariate logistic regression analysis, self-reported SNP was significantly associated with, age, marital status, monthly salary, working organization, previous smoker, habit of drinking alcohol, doing physical exercise, teaching experience, teaching hour per week, using static head down posture, elevated arm over the shoulder, prolonged sitting, diabetes mellitus, hypertension and respiratory diseases. However, in the multivariate logistic regression analysis, self-reported SNP was associated significantly (*p* < 0.05) with regular physical exercise (OR = 0.18, 95% CI: 0.08–0.42), teaching experience (OR = 2.85, 95% CI: 1.09–7.42), static head down posture (OR = 2.26, 95% CI: 1.55–3.33), elevated arm over the shoulder (OR = 2.71, 95% CI: 1.86–3.95), prolonged sitting (OR = 1.50,95% CI: 1.02–2.23) and hypertension (OR = 2.18, 95% CI: 1.24–3.82) (Table 4).

**Table 4:** Factors associated with SNP among school teachers in Gondar town with bivariate and multivariate logistic regression analysis, 2017 (*N* = 754)

	SNP		COR (95% CI)	AOR (95% CI)	*p*-value
	YES	NO			
Age					
<=30	35	82	1		
31-40	123	124	2.38 (1.49, 3.79)	1.13 (0.56, 2.29)	0.737
41-50	142	73	4.67 (2.89, 7.58)	1.66 (0.71, 3.91)	0.245
>50	132	41	7.73 (4.56, 13.09)	1.82 (0.67, 5.05)	0.236
Marital status					
Single	26	70	1		
Married	383	241	4.28 (2.65, 6.90)	1.59 (0.85, 3.01)	0.148
Other	23	11	5.63 (2.41, 13.14)	2.44 (0.89, 6.65)	0.082
Salary					
2000-4000	57	98	1		
4001-6000	322	195	2.84 (1.96, 4.12)	1.01 (0.58, 1.75)	0.976
>6000	53	29	3.14 (1.79, 5.49)	0.72 (0.33, 1.57)	0.410
Organization					
Public	374	255	1.69 (1.15, 2.49)	0.82 (0.50, 1.36)	0.447
Private	58	67	1		
Previous smoker					
Yes	43	15	2.26 (1.23, 4.15)	1.41 (0.68, 2.89)	0.347
No	389	307	1		
Alcohol consumer					
Yes	40	16	1.95 (1.07, 3.55)	1.33 (0.64, 2.78)	0.450
No	392	306	1		
Physical exercise					
Yes	9	45	0.13 (0.06, 0.27)	**0.18 (0.08, 0.42)**	0.000*
No	423	277	1		
Teaching experience					
2-9	47	104	1		
10-19	161	215	2.94 (1.94, 4.47)	**1.96 (1.03, 3.69)**	0.039*
20-29	102	61	3.70 (2.32, 5.91)	1.58 (0.70, 3.57)	0.268
>=30	122	36	7.49 (4.52, 12.45)	**2.85 (1.09, 7.42)**	0.032*
Teaching hours/week					
<=30	365	250	1		
>30	67	72	1.57 (1.09, 2.27)	0.66 (0.43, 1.03)	0.067
Static head down posture					
Yes	342	186	2.78 (2.02, 3.83)	**2.26 (1.55, 3.33)**	0.000*
No	90	136	1		
Elevated arm over shoulder					
Yes	342	181	2.96 (2.15, 4.08)	**2.71 (1.86, 3.95)**	0.000*
No	90	141	1		
Prolonged sitting					
Yes	161	86	1.63 (1.19, 2.23)	**1.50 (1.02, 2.23)**	0.041*
No	271	236	1		
Diabetes mellitus					
Yes	84	29	2.44 (1.55, 3.82)	1.22 (0.70, 2.11)	0.479
No	348	293	1		
Hypertension					
Yes	105	23	4.17 (2.59, 6.73)	**2.18 (1.24, 3.82)**	0.006*
No	327	299	1		
Respiratory disease					
Yes	49	16	2.45 (1.36, 4.39)	1.58 (0.80, 3.12)	0.184
No	383	306	1		

*=*p*-value is <0.05, *COR* crud odds ratio, *CI* confidence interval, *AOR* adjusted odds ratio, *SNP* shoulder neck pain

## Discussion

This is the preliminary study that investigated the prevalence of shoulder and/or neck pain and the association of individual, health, and occupational characteristics among primary and secondary school teachers was assessed. The previous 12 months self-reported prevalence of shoulder and/or neck pain among primary and secondary school teachers was 57.3% (95% CI: 53.4–61.0), the results indicated that SNP is a common occupational health problem among school teachers and suggestive of some specific participant, health, and working condition may increase the prevalence rate among Ethiopian school teachers. Though, studies that reported prevalence rate of SNP are distinctively scant. [12, 16, 21] The findings of this study is similar to a study done among Malaysian school teachers reporting 60% prevalence [22]**.** However, the prevalence of this study is higher than the studies conducted in China (48.7%) [12], Saudi Arabia (45.2%) [23], Japan (35.4%) [24] and Brazil (31.6%) [25]**.** This difference observed in prevalence rate of SNP could due to the differences in educational system, study design, the facility provided for the teachers at their institution or social, cultural, and economic differences between Ethiopia and other countries., In addition, working hours, the way in which work was organized, children teacher ratio, and the protective factors involved contribute to the differences observed in comparison to the present study.

The prevalence reported in this study was lower than the prevalence reported in studies conducted in India (73.5%) [26] and Taiwan (63.4%) [27]**.** The possible reason for the observed dissimilarity could be due to difference in study design, study population, and teaching conditions, the Indian study included six school levels (starting from primary to technical schools) of teacher, and the Taiwan study included teachers by purposive sampling and used mailing for data collection from special education schools. Special education teachers work with children who have a variety of mental, emotional, physical, and learning disabilities which is demanding with special challenges.

The present study also indicated that habit of doing physical exercise has a positive effect on the shoulder and/or neck pain among school teachers and the risk of developing SNP reduced by 82% as compared to those with no exercise.. This result was consistent with studies done in Turkey [28], China [12] and Botswana [9]. The possible reasons may be exercises can improve strength, flexibility, pain threshold, and makes muscles and ligaments stronger to support the neck and shoulder alignment for optimal functioning and prevents injury [9, 29].

Studies conducted in Botswana [9], Egypt [30], and Saudi Arabia [23] reported an association between teaching experience and the prevalence of SNP. Similarly in the present study, there is a significant association between SNP and increasing year of teaching experience. Teachers with more than 30 years of teaching experience were 2.85 times more likely to develop SNP than those with less than 10 years of teaching experience and those who have 10–19 years teaching experience were 1.96 times more likely to develop SNP when compared to those with less than 10 years teaching experience. The possible reasons could be effect of aging, age-related degenerative changes, declined tissue healing, thinning of cartilage, and cumulative trauma to neck and shoulder structures due to workload. Most available studies reported an association of age and length of employment with SNP. More so, in this study and Ethiopia, teachers start teaching at an early age after completion of a diploma or bachelor degree. Hence, the possibility of a higher length of employment, longer exposure to occupational- related hazards, and eventually job-related musculo-skeletal disorders at much younger age compared with many other countries. [4, 5, 21]

Teachers spending more than 2 h per day with a static head down posture for reading, paper evaluations, scoring, and preparing class works is associated with shoulder and/or neck pain in the present study. This result was similar to a study in China [31]**.** The reason might be sustained head down posture could result in straining of neck structures causing discomforts, muscle stiffness or tightness around cervico-brachial region eventually pain [29, 32] . In addition, the student-teachers ratio was higher, 81.2% of teachers included in this study had more than 40 students in a class.

In this study elevated arm (arm above shoulder) was significantly associated with shoulder and/ or neck pain. Elevating arm above shoulder during writing on board was 2.71 times more likely to develop a chance of SNP compared to not elevating arm above shoulder. This result was in similar to studies done in Botswana [9] and Egypt [30]**.** Possible reasons could be, working with raised arms above unsupported shoulder for a long time can cause friction, tension, and strain over cervico-brachial regions [27, 28, 33] . This is normally observed in teachers’ daily routine to write on the board. This mechanism can cause teachers to develop discomfort in the cervico-brachial regions, which is even made worse by daily overwork and less rest time.

The other work-related factor associated with shoulder and/or neck pain was prolonged sitting. Teachers those who reported prolonged sitting (>4Hr.) were 1.50 times more likely to develop SNP. This result was comparable with a study done in China among school teachers [12] **.** The possible reason might be teachers sitting on uncomfortable chair and table tends to develop stiffness and discomfort. In this study, more than 3/4th (82.2%) of teachers were reported as they were not using comfortable support during sitting.

In this study, teachers those who self-reported to have hypertension as diagnosed by physician were significantly associated with shoulder and/or neck pain. Teachers with self-reported hypertension were 2.18 times more likely to develop SNP compared to those who had no hypertension. This result is consistent with a study done in Malaysia among secondary school teachers [22] **.** Hypertension by itself due to more pressure in the vessels of cervical region or along with postural syndrome might result in shoulder and neck pain. Few physicians even report that neck musculatures could play a crucial role in controlling blood pressure. Hence, the symptoms of hypertension by itself could result in shoulder and neck pain or it could even worsen the severity of pain during repetitive daily school tasks. This study has provided an insight into the frequency of shoulder and neck pain among the school teaching community in a resource-limited country. Though 88.9% of the calculated sample was reached, with the higher than anticipated prevalence being reached post hoc power analysis showed 100% power.

### Study limitations

Considering the benefits of future research there are few limitations to be mentioned. Psychological factors, postural assessment, home and job site evaluations were not considered. These confounders could lead to a possible variation in the estimation of association among SNP and other variables. The cross-sectional nature does not allow inferring of causality and effect. This study also included both primary and secondary school teachers with variable working demands which might be a possible source of heterogeneity in the study sample. Hence the findings of this study should be interpreted with caution. Future studies should address these concerns and determine causality and effect among school teachers. Nevertheless, this is a preliminary work to provide a powered insight into the burden of shoulder and /neck pain among school teachers in Ethiopia.

## Conclusion

In conclusion, this study showed that SNP is a common occupational-related health problem among both primary and secondary school teachers in Ethiopia. Work-related, and health characteristics like posture, health and working conditions such as teaching experience, using static head down posture, elevated arm over the shoulder, prolonged sitting during and hypertension were associated with shoulder and neck pain. Whereas engaging in regular physical exercise has a protective effect for shoulder pain. Related factors that may help explain SNP among school teachers should be explored by future studies. The school authorities are recommended to provide facilities to enhance physical activity among school teachers and also provide adjustable board and classroom materials. School teachers are recommended to develop awareness of related health hazards and encourage the habit of regular physical exercise.

## Additional file

Additional file 1: Ethiopian school teacher shoulder/and neck pain questionnaire. This questionnaire has five categories of independent variables and one category for the dependent variable. A questionnaire was structured from similar studies for socio-demographic, behavioral, work related physical factors, comorbidities, and psychosocial factors. Tools used to assess shoulder/neck pain was adapted from the standardized Nordic questionnaire and modified to local context. (PDF 1182 kb)

## Abbreviations

AOR: Adjusted odds ratio
BMI: Body mass index
COR: Crude odds ratio
LMIC’s: Low-Middle Income Countries
MSD: Musculo-skeletal disorder
MSP: Musculoskeletal pain
OR: Odd’s ratio
PR: Prevalence ratio
SD: Standard deviation
SNP: Shoulder and/or neck pain
ULD: Upper limb disorder
WHO: World Health Organization
WRMSP: Work related musculo-skeletal pain
