# Supplementary material for: Burden of shoulder and/neck pain among school teachers in Ethiopia
Source: BMC Musculoskelet Disord. 2019 Jan 10;20:18. doi: 10.1186/s12891-019-2397-3 (PMC6329165; doi:10.1186/s12891-019-2397-3)
Supplement: Supplementary file 1 — Ethiopian school teacher shoulder/and neck pain questionnaire. This questionnaire has five categories of independent variables and one category for the dependent variable. A questionnaire was structured from similar studies for socio-demographic, behavioral, work related physical factors, comorbidities, and psychosocial factors. Tools used to assess shoulder/neck pain was adapted from the standardized Nordic questionnaire and modified to local context. (PDF 1182 kb) [file 12891_2019_2397_MOESM1_ESM.pdf]

Appendix 1: Ethiopian school teacher shoulder/and neck pain [EST-SNP] Questionnaire

English version Questionnaire **Date:**

**Socio-demographic information:**		**Questionnaire unique ID:**	
**Data collector ID:**		**Response**	**Code**
101	Sex		D1
	If female aye you pregnant?	1. Yes 2. No	
102	Age		D2
103	Educational status	1. Certificate<br>2. Diploma<br>3. Degree<br>4. Master	D3
104	Marital status	1. Single<br>2. Married<br>3. Divorced<br>4. Separated<br>5. Widowed	D4
105	Handedness	1. right<br>2. Left	D5
106	Monthly income		D6
107	Height		
108	Weight		
109	Body mass index		D7
110	School level	1. Elementary<br>2. High school	D8
111	Working organization	1. Private<br>2. Governmental	D9

Behavioral factors			
201	How would you describe your smoking habit?	1. Previous occasional smoker<br>2. Previous daily smoker<br>3. Non smoker<br>4. Current occasional smoker<br>5. Current occasional smoker	B1
202	Do you consume alcohol?	1. Yes 2. No	B2
	If yes how many bottles/ml in a week?		
203	Do you do physical exercise	1. Yes 2. No	B3
	If yes how many minuets in a week?		
**Shoulder/neck Pain**			
**301**	Have you ever had discomfort/pain around your shoulder/neck during last 12 month?	1. Yes<br>2. No	P1
**302**	If yes on which part of body you feel the pain?	1. Both (shoulder/neck)<br>2. Shoulder only<br>3. Neck only<br>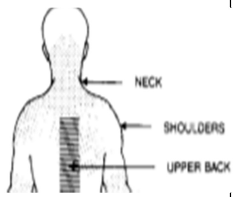	P2
**303**	What is the onset of your pain?	1. Gradual 2. Sudden	P3
**304**	For how long do you feel this pain?	1. Less than 7 days<br>2. 7 days -7 weeks	P4

		3. 8-12 weeks<br>4. More than 12 weeks	
**305**	How severe is your pain?	___________________<br>___Mark on the 10cm line	P5
**306**	Do you have absenteeism from your work due to pain?	1. Yes 2. No	P6
**307**	Does the pain go in to your arm or hand?	1. Yes 2. No	P7
**308**	Have you sought consultation with any doctor/Physiotherapists for your pain?	1. Yes 2. No	P8
**Work related physical factors**			
**401**	Work experience	…………years	W1
**402**	How many working hours of class do you have in a week?	______ mint its	W2
**403**	How many students you teach in one class?	1. 25-30<br>2. 30- 40<br>3. >40	W3
**404**	Have you ever experienced static head down posture more than 2- hours per day while correcting students work and other activities?	1. Yes 2. No	W4
**405**	Have you ever experienced elevated arm above your shoulder more than 2- hours per day while writing on blackboard?	1. Yes 2. No	W5
**406**	Have you ever experienced standing position more than 2- hours per day in a class?	1. Yes 2. No	W6
**407**	Have you ever experienced sitting position more than	1. Yes 2. No	W7

	4- hours per day in a class?		
**408**	Do you have comfortable back support during sitting?	1. Yes 2. No	W8
**Comorbidities**			
**501**	Do you have diagnosed diabetes mellitus?	1. Yes 2. No	C1
**502**	Do you have diagnosed hypertension?	1. Yes 2. No	C2
**503**	Do you have diagnosed respiratory diseases (asthma)?	1. Yes 2. No	C3
**504**	Have you ever had surgery around your shoulder/ neck area before 3- month?	1. Yes 2. No	C4
**505**	Have you ever had trauma around your shoulder/ neck area before 3- month?	1. Yes 2. No	C5

**Psychosocial factors**						
601.	Psychological job demand	To a very large extent	To a large extent	Somewhat	To a small extent	To a very small extent
602.	Supervisor support	Always	Often	Sometimes	Seldom	Never/hardly ever)
603.	Social support	Always	Often	Sometimes	Seldom	Never/hardly ever)
604.	Co-worker support	Always	Often	Sometimes	Seldom	Never/hardly ever)
605.	Job satisfaction	To a very large extent	To a large extent	Somewhat	To a small extent	To a very small extent

Thank you for your participation!!!

Amharic version questionnaire

**ማህበራዊና ስነ-ህዝብ መረጃ**

		የመልስ አማራጭ	መ/ቁ
101	ጾታ	1. ወንድ 2. ሴት	D1
	ሴት ከሆኑ እርጉዝ ነዎት	1. አዎ 2. አይደለሁም	
102	ዕድሜ		D2
103	የትምህርት ደረጃ	1. ሰርቲፊኬት<br>2. ድፕሎማ<br>3. ድግሪ<br>4. ማስተርስ	D3
104	የጋብቻ ሁኔታ	1. ያላገባ;ች<br>2. ያገባ/ች<br>3. የፈታ;ች<br>4. የሞተችበት/የሞተባት	D4
105	ብዙ ጊዜ የሚትጠቀምበት/ሚበት እጅ	1. ቀኝ 2. ግራ	D5
106	ወርሃዊ ክፍያ		D6
107	ቁመትህ/ሽ		
108	ክብደትህ/ሽ		
109	የሰዉነትህ/ተሽ ምልከታ		D7
110	የምታሰተምርበት የትምህርት ደረጃ	1. አንደኛ ደረጃ<br>2. ሁለተኛ ደረጃ	D8
111	የምትሰራበት ምስራቤት	1. የግል 2. የመንግስት	D9

የግል ልማድ የሚመለከት መረጃ			
201	ስጋራ የሚጨስ ልማድ አለህ/ሽ	1. አልፎ አልፎ አጨስ	B1

		ነበር<br>2. በየቀኑ አጨስ ነበር<br>3. አላጨስም<br>4. አልፎ አልፎ አጨሳለሁ<br>5. በየቀኑ አጨሳለሁ	
202	አልኮል ትጠጣለህ/ሽ	1. አዎ 2. አላጠጣም	B2
	አዎ ከሆነ በሳምንት ምን ያኽል		
203	የአካል ብቃት እንቅስቃሴ ትሰራለህ/ሽ	1. አዎ 2. አልሰራም	B3
	አዎ ከሆነ በሳምንት ምን ያህል ደቂቃ		
**የትክሻና/አንገት ህመም መረጃ**			
**301**	ባለፉት 12 ወራት በትክሻህ/ሽና አንገትህ/ሽ አከባቢ ህመም ወይም ጥሩ ያልሆነ ስሜት ተሰምቶህ/ሽ ያውቃል	1. አዎ 2. አያዉቅም	P1
**302**	መልስህ አዎ ከሆነ የትኛው የሰዉነት አከባቢ ነው<br>(በስዕሉ ላይ መጠቆም ይቻላል)	1.ትክሻና አንገት አከባቢ<br>2.ትክሻ አከባቢ ብቻ<br>3.አንገት አከባቢ ብቻ<br>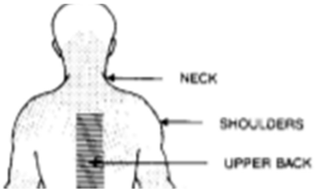	P2
**303**	የህመሙ አጀማመር እነዴት ነበር	1.በሂደት 2. በድንገት	P3
**304**	ህመሙ ለምን ያኽል ጊዜ ተሰምቶሃል/ሻል	1. ከ7 ቀን በታች<br>2.ከ7 ቀን እስከ 7 ሳምንት<br>3. 8-12 ሳምንት<br>4. ከ12 ሳምንት በላይ	P4
**305**	የህመሙ መጠን ምን ያኽል ይሆናል	________	P5

		ከ1 እስከ 10 ስገለጽ	
**306**	በህመሙ ምክንያት ከስራ ቀርተህ/ሽ ታዉቃለህ	1.አዎ 2. አላዉቅም	P6
**307**	ህመሙ ወደ ክንድህ/ሽና አጅህ/ሽ አከባቢ ይደርሳል	1. አዎ 2. አይደርስም	P7
**308**	ለህመምህ/ሽ የጤና ባለሞያ/ሓኪም አማክረህ ታዉቃለህ	1. አዎ 2.አላዉቅም	P8
**ከስራ ጋራ የተያያዘ መረጃ**			
**401**	በመምህርነት ምን ያኽል ገዜ አገልግለሃል/ሻል	…………ዓመት	W1
**402**	በሳምንት ምን ያኽል ሰዓት ታሰተምራለህ/ሽ	_______ ደቂቃ	W2
**403**	በአንድ ክፍል ምን ያህል ተማሪ አለ	1. 25-30<br>2. 30- 40<br>3. >40	W3
**404**	የተማሪዎች ስራ ለማረም፣ ዕቅድ ለማዘጋጀት ወይም በምታሰተም/ሚርበት ጊዜ በቀን አንገትህ/ሽ አጥፈህ/ሽ በአማካኝ ከሁለት ሰዓት በላይ ትቆያለህ/ሽ	1. አዎ 2. አልቆይም	W4
**405**	ሰሌዳ ላይ በምትጽፍበት ጊዜ በቀን ክንድህ/ሽ ከትክሻህ/ሽ በላይ አድርገህ በአማካኝ ከሁለት ሰዓት በላይ ትቆያለህ/ሽ	1. አዎ 2. አልቆይም	W5
**406**	በምታሰተምርበት ጊዜ በቀን ከሁለት ስዓት በላይ ትቆማለህ/ሽ	1.አዎ 2. አልቆምም	W6
**407**	በምታሰተምርበት ጊዜ በቀን ከአራት ስዓት በላይ ትቀመጣለህ/ሽ	1.አዎ 2. አልቀመጥም	W7
**408**	በምትቀመጥበት ጊዜ አመቺ የሆነ የወገብ ድጋፍ ያለዉ ወንበር ትጠቀማለህ	1.አዎ 2. አልጠቀምም	W8
**ተ∏ዳኝ የጤና ችግሮች መረጃ**			
**501**	በህክምና የታወቀ የሰ≤ር ህመም አለብህ/ሽ	1.አዎ 2. የለኝም	C1
**502**	በህክምና የታወቀ የደም ገፊት አለብህ/ሽ	1.አዎ 2. የለኝም	C2
**503**	በህክምና የታወቀ የመተንፈስ ችግር አለብህ/ሽ	1. .አዎ 2. የለኝም	C3
**504**	ከሦስት ወር በፊት በትክሻህና አነገትህ አከባቢ ቀዶ ጥገና ተሰርቶልህ ያዉቃል	1. .አዎ 2. አያዉቅም	C4

**504**	ከሦስት ወር በፊት በትክሻህና አነገትህ አከባቢ አደጋ አጋጥሞህ ያዉቃል	1. .አዎ 2. አያዉቅም	C5

**ስነ-ልቦናዊና ማህበራዊ መረጃ**						
601.	በምታስተምርበት አከባቢ ባለ የመማር ማስተማር ስነልቦናዊ ዝግጅትና የሞራል ድጋፍ ምን ያኽል ትረካለህ/ሽ	እጅግ በጣም እረካለሁ	በጣም እረካለሁ	በዉሱን መጠን እረካለሁ	በጣም በትንሹ እረካለሁ	እጅግ በጣም በተነሹ እረካለሁ
602.	አለቃህ ምን ያኽል ይረዳሃል	ሁሉ ጊዜ	ብዙ ጊዜ	አንዳንድ ጊዜ	እምብዛም	አያግዘኝም
603.	በምታስተምርበት አከባቢ ያለው ማህበረ ሰብ ምን ያኽል ይረዳሃል	ሁሉ ጊዜ	ብዙ ጊዜ	አንዳንድ ጊዜ	እምብዛም	አያግዘኝም
604.	የስራ ባልደረባህ/ሽ ምን ያኽል ያግዝሃል /ያግዝሻል	ሁሉ ጊዜ	ብዙ ጊዜ	አንዳንድ ጊዜ	እምብዛም	አያግዘኝም
605.	በስራህ/ሽ ምን ያኽል ትረካለህ/ሽ	እጅግ በጣም እረካለሁ	በጣም እረካለሁ	በዉሱን መጠን እረካለሁ	በጣም በትንሹ እረካለሁ	እጅግ በጣም በተነሹ እረካለሁ
